# Seasonal monitoring of forage C:N:ADF ratio in natural rangeland using remote sensing data

**DOI:** 10.1007/s10661-024-13579-x

**Published:** 2025-01-06

**Authors:** Monde Rapiya, Abel Ramoelo, Wayne Truter

**Affiliations:** 1 Department of Plant and Soil Sciences, University of Pretoria, Hatfield, 0001 Pretoria South Africa; 2 Centre for Environmental Studies, Department of Geography, Geoinformatics and Meteorology, University of Pretoria, Hatfield, 0001 Pretoria South Africa

**Keywords:** Forage nutrients, Mesic rangelands, Sentinel-1, Sentinel-2

## Abstract

In recent decades, natural rangelands have emerged as vital sources of livelihood and ecological services, particularly in Southern Africa, supporting communities in developing regions. However, the escalating global demand for food, driven by a growing human population, has led to the extensive expansion of cultivated areas, resulting in continuous nutrient leaching in rangelands. To ensure the long-term viability of these ecosystems, there is a need to develop effective approaches for managing and monitoring the seasonality of forage quality. This study aims to achieve this by utilizing multispectral Sentinel-1 (S1) and Sentinel-2 (S2) data to monitor the seasonal distribution and occurrence of carbon (C), nitrogen (N), acid detergent fiber (ADF), and the (C:N:ADF) ratio in mesic rangelands. Six sites were randomly selected from Welgevonden and Hoogland private game reserves in Limpopo, South Africa, representing varying vegetation cover and standing biomass. Transects, each with ten fixed sample sites (30 × 30 m) characterized by homogeneous vegetation, were established. The grass samples and aboveground biomass were collected during each season and analyzed for biochemical parameters using a near-infrared spectroscopy (NIRS) machine. S1 and S2 data from Google Earth Engine (GEE) were employed, and the random forest (RF) modelling algorithm revealed significant seasonality impacts on the distribution of forage C:N:ADF ratios. The study demonstrates that integrating S1 and S2 data enhances the estimation of forage nutrients. This study offers valuable insights for a diverse range of stakeholders, including ecologists, resource managers, farmers, and park managers. By giving an understanding of nutrient limitations and facilitating a deeper understanding of resource availability and animal distribution in rangelands, this research serves as a crucial tool for informed decision-making and sustainable management practices.

## Introduction

In recent decades, there has been a growing recognition of the vital role that natural rangelands play in the livelihoods and ecological services of people in developing regions, particularly in Southern Africa (McGranahan & Kirkman, 2013). These areas heavily rely on emerging or resource-poor farmers for livestock production, and natural rangelands serve as their primary source of grazing land. The grasses in these rangelands provide essential nutrients for animal production, making them crucial for the sustainability of rural livelihoods. Historically, various management interventions have been implemented to monitor and improve resource stewardship in rangelands, aiming to protect these natural resources (Scholes, 2009). However, rangelands worldwide continue to face continuous threats from human actions, such as land-use change and overutilization, as well as environmental challenges like climate change and droughts (Narouei et al., 2022). With the world’s population steadily increasing, the demand for food has also risen significantly. This has led to an expansion of cultivated areas, causing soil erosion and nutrient depletion. Similarly, the reduction of rangelands has resulted in vegetation loss and runoff, contributing to the degradation of these critical resources (Palmer & Bennett, 2013).

As a result, it has been reported that nearly 40% of the world’s rangelands are degraded, posing a threat to roughly half of the global GDP, which amounts to $44 trillion annually (UNCCD, 2022). This situation is expected to worsen by 2025 if serious interventions are not put in place to monitor vegetation in rangelands (UNCCD, 2022). Addressing these extensive and serious constraints is essential for improving the contribution of livestock to rural livelihoods (Vetter, 2013). Fluctuations in forage quality, particularly the concentrations of nitrogen (N) and fiber, have significant implications for livestock production and pastoralists’ livelihoods. These fluctuations can reduce the nutrient content and nutritive value of forage, negatively impacting animal production (Ravhuhali et al., 2022). Therefore, understanding these fluctuations is critical to mitigating their effects on livestock production and pastoral livelihood sustainability. Developing effective approaches to manage and monitor the seasonality of forage quality is necessary to support the long-term viability of rangelands.

Regional mapping of grass biochemical parameters, including forage carbon (C), forage nitrogen (N), and forage digestibility (acid detergent fiber (ADF)), has been identified as a key indicator of grass quality, quantity, and palatability. These parameters are crucial for understanding forage nutrition conditions and feed value and play a significant role in vegetation ontogeny. Carbon content (C) reflects biomass accumulation in vegetation, while forage digestibility influences the energy animals can extract from their forage for production (Hungate et al., 2013; Vethathirri et al., 2021). Nitrogen (N) is a critical element in forming protein, which is an essential nutrient for grazing animals. N content significantly affects the quality of forage available in rangeland biomass, and it plays a crucial role in determining plant growth and productivity, thereby affecting the nutritional value of forage (Gao et al., 2020a; Zhang et al., 2023). Acid detergent fiber (ADF) is a crucial parameter for assessing forage quality as it quantifies the presence of indigestible plant fibers like cellulose, lignin, and silica. ADF directly relates to forage digestibility, which is essential for animal health and productivity. Precise determination of ADF levels in forage can provide valuable information for optimizing animal nutrition and enhancing animal performance (Amiri, 2012; Dasci et al., 2010). Understanding all three forage components provides essential data on both forage quantity and quality as well as forage digestibility, which is crucial for sustainable planning and management of feeding patterns and the distribution of grazing animals in rangelands (Gao et al., 2020a; Ramoelo et al., 2012).

The quantification of C, N, and ADF concentrations as a means of determining forage production is significant. Surprisingly, there is no scientific study in the literature about the forage C:N:ADF ratio in rangelands, despite its potential to provide a balance of C, N, and ADF availability. This ratio can assist in precise data collection about forage quality, quantity, palatability distribution, and nutrient limitations in natural rangelands, helping determine animal distribution and grazing interventions. Some studies have noted the usefulness and reliability of the foliar ratio as an indicator of nutrient limitation (Gao et al., 2020b; Prins & van Langevelde, 2008; Ramoelo et al., 2012). Understanding the C:N:ADF ratio can assist researchers and practitioners in optimizing vegetation to improve the efficiency and sustainability of animal production.

Remote sensing technology, such as satellite imagery, has played a crucial role in mapping grass biochemical parameters over large areas (Mutanga & Kumar, 2007; Ramoelo et al., 2012). Unlike traditional approaches, these tools offer cost-effective and efficient means of collecting data on vegetation and grass quality parameters, which can inform decision-making processes related to land use and grazing management (Wang et al., 2020; Yang et al., 2017). However, remote sensing approaches have faced challenges such as the atmospheric effect, soil background, sensitivity, and saturation of vegetation indices like the Normalized Difference Vegetation Index (NDVI) at peak production (Gao et al., 2020b; Mutanga & Skidmore, 2004). Nevertheless, the development of new remote sensing tools with high spatial resolution and red-edge wavelength has overcome these limitations during the measurement of biochemical parameters (Gao et al., 2020a; Ramoelo et al., 2013). These new tools are less sensitive to atmospheric effects and soil background and can measure the vegetation reflectance spectrum up to 680–750 nm, providing more precise data on vegetation characteristics. These improvements have enabled accurate data acquisition for biophysical and biochemical parameters without significant restrictions or limitations (Gao et al., 2020a; Mutanga & Skidmore, 2004).

Remarkably, there is no scientific study in the literature that has monitored and assessed the forage C:N:ADF ratio using remote sensing data in tropical rangelands. A study by Gao et al. (2020b) explored the potential use of hyperspectral data to estimate the carbon: nitrogen ratio in the grasslands of the Tibetan Plateau. Therefore, the primary aim of this study is to monitor the spatial and temporal variation of forage C:N:ADF ratio in mesic rangelands using multispectral Sentinel-1 (S1) and Sentinel-2 (S2) data. Secondly, the study aims to develop predictive seasonal models for estimating the spatial distribution of forage C:N:ADF ratio in mesic rangelands. By utilizing Sentinel-1 and Sentinel-2 data, the study seeks to determine the capability of S1 and S2, both individually and in combination, in estimating the seasonal forage quality. Furthermore, the changes in biochemical content and structure of vegetation throughout the seasons lead to visible spectral changes in vegetation. For example, as vegetation dries and becomes senescent, there is a shift in the red-edge shoulder and an increase in the importance of shortwave infrared (SWIR) features associated with lignin and cellulose. These photosensitive properties can potentially be utilized for predicting and mapping forage nutrients during each season (Asner et al., 2000; Kokaly & Clark, 1999). Therefore, assessing spectral variables of importance from these sensors during monitoring of seasonal forage quality is crucial as different spectral variables are associated with specific wavelengths of light absorbed, reflected, or transmitted by vegetation (Durante et al., 2014). This information can aid in understanding different physiological and structural characteristics of vegetation, including nitrogen content and biomass (Niinemets et al., 2015). These datasets offer valuable information for monitoring biochemical parameters and accurately estimating forage quality in the area. This data is crucial for sustainable planning and managing livestock and wildlife grazing (Gao et al., 2020a; Raab et al., 2020; Ramoelo et al., 2012; Tessier & Raynal, 2003; Zhang et al., 2023).

## Material and methods

### Study area

**Fig. 1:**
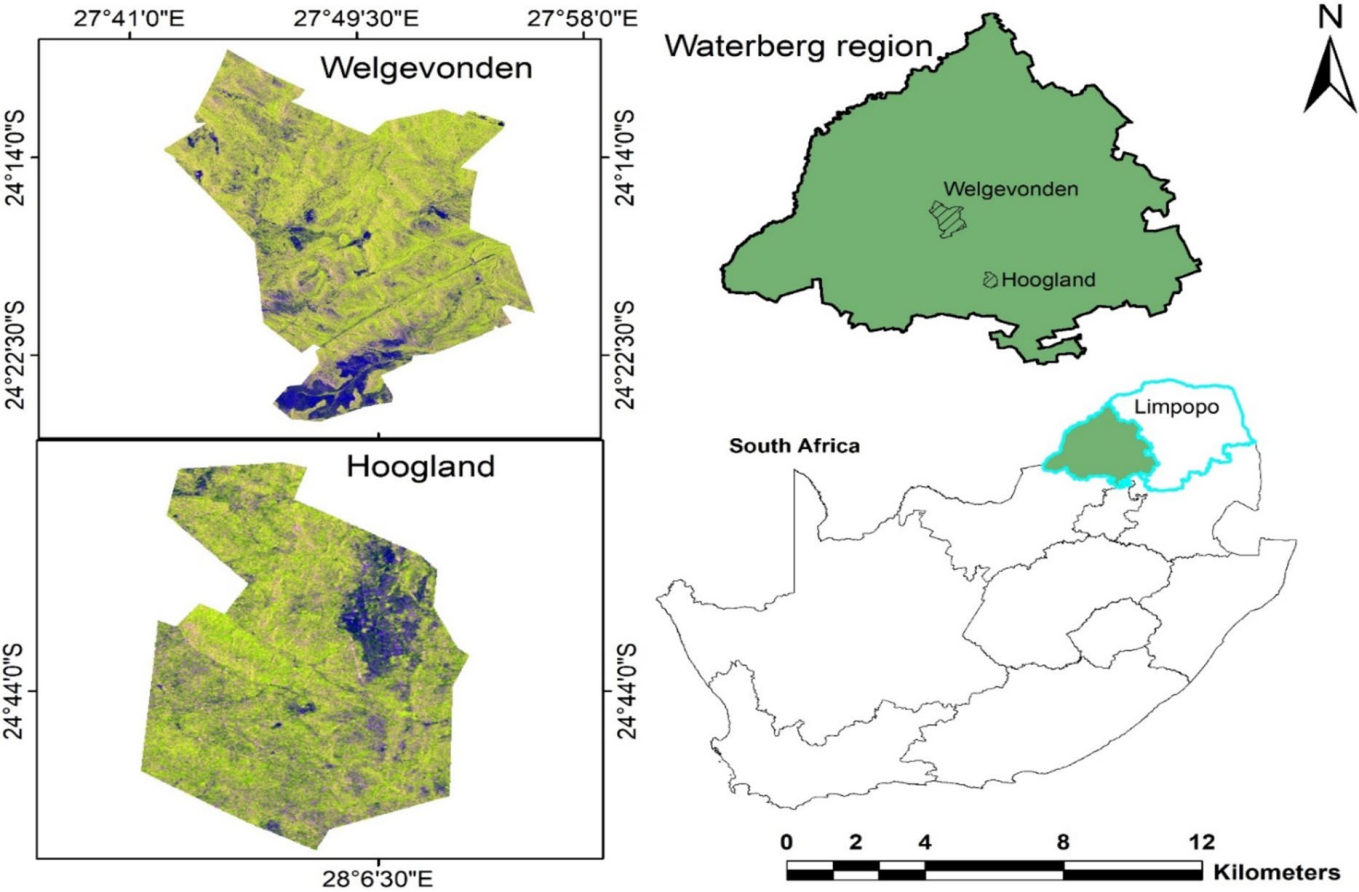
Illustration with two study sites in the Waterberg region of Limpopo province, South Africa. Welgevonden game reserve (**a**) and Hoogland game reserve (**b**)

This study was conducted in two privately owned game reserves: The Welgevonden game reserve (24° 10′ to 24° 25′ S; 27° 45′ to 27° 56′ E) and the Hoogland game (24° 43′ to 20.8′ S; 28° 07′ to 48.7″ E) in the north of the Bushveld Basin of Waterberg Estate, Limpopo Province, South Africa (Fig. 1). The altitude of the study area ranges between 300 and 900 m above mean sea level. The climate is predominantly low to hot (“mean minimum temperature is 14.4 ℃, and mean maximum temperature is 44.9 ℃”) in summer and receives average annual rainfall of 790–1174 mm with mean annual evaporation comprised between 1750 and 1900 mm (Institute of Soil, Climate and Water, 1999; Nesamvuni et al., 2003). The region is comprised of sandstone and a sequence of clastic, sedimentary rocks (Callaghan, 1987; Frost et al., 2001). The sandstone results to well-drained rough sandy soils with modest clay concentration (1.7 to 2.9%) (Kilian, 2003). The vegetation type is mostly “sourveld,” which covers Lowveld Sour Bushveld, patches of the North-Eastern Sourveld in the North, and South-Eastern Sourveld in the South, as described by Acocks (1988). Common herbaceous species, which are the symbol of sourveld, include “*Schizachyrium sanguineum*, *Schizachyrium Jeffreysii, Elionurus muticus, Loudetia simplex, Diheteropogon amplectens, Hyperthelia dissoluta, Trachypogon spicatus, Panicum natalense, Brachiara nigropedata, Eragrostis curvula, Eragrostis superba, Sporobolus pectinatus, Heteropogon contortus, Aristida spp., Pogonarthria squarrosa, Melinis repens* and *Urelytrum agropyroides*” (Kilian, 2006). For the last 30 years, the Welgevonden game reserve has been naturally managed and protected from external factors (such as wildfire) that can lead to completely removing vegetation. Despite their low fire risk due to high soil moisture (Grant & Groenevelt*, *2015), Hoogland was impacted by a severe wildfire in 2017. Both reserves were naturally grazed by wildlife and hold significant importance for biodiversity conservation, as they support ecological processes, preserve genetic diversity, provide essential ecosystem services, and serve as indicators of ecosystem health (Rawat & Agarwal, 2015).

### Data collection

**Fig. 2:**
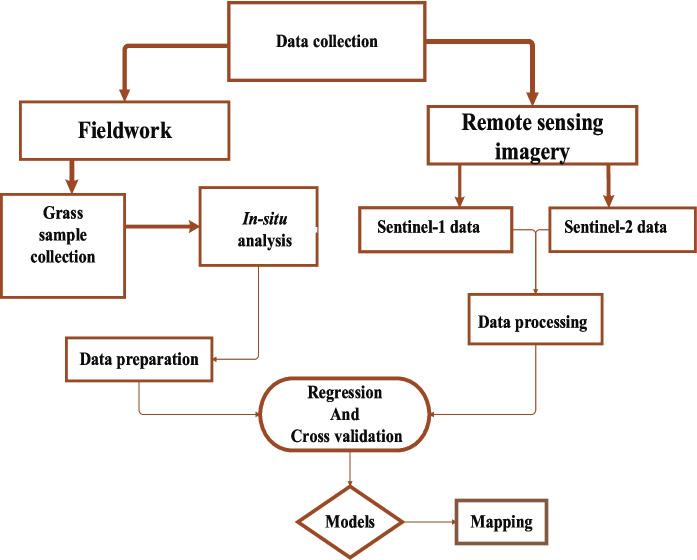
Flow chart of conceptualizing the procedures followed in this study

The study conducted field data collection over a period of 2 years, from November 2020 to March 2022, encompassing three distinct seasons early summer (November–December 2020), winter (July–August 2021), and late summer (March 2022). Six yielded sites were randomly selected from the Welgevonden and Hoogland reserves, each with varying vegetation cover and standing biomass to ensure representative sampling. Within each area, transects were established using a combination of systematic placement and purposive sampling plots. Each transect was then subdivided into ten 30 × 30 m plots with “homogeneous vegetation” to capture variability. A total of 10 quadrats of 1 m^2^ (a total of 180) were randomly placed in each plot and then moved in each sampling time to avoid re-sampling. The grass was clipped using scissors at a theoretical height of 5–8 cm above the ground to ensure consistency in sampling. The collected grass samples were then dried in an oven at 70 °C for 48 h to prepare them for further chemical analysis. This severe field data collection process ensured a thorough and reliable assessment of the ecological parameters and enabled the researchers to draw robust conclusions from their study (Fig. 2).

### Conversion of aboveground biomass to the amount of carbon

The conversion of above-ground biomass (kg/ha) to stored carbon values is a widely used method to evaluate the amount of carbon stored in terrestrial ecosystems. This approach relies on a conversion factor, representing the quantity of carbon stored in a given biomass. One of the most commonly used conversion factors is 47%, which is based on the assumption that dry terrestrial biomass has an average carbon content of 47%. The assumption of a 47% conversion factor is supported by IPCC (2006), for the investigation of the carbon concentration of heterogeneous herbaceous vegetation components (savanna). Hence, this study used a 0.47 conversion factor for carbon calculation from the field aboveground biomass of heterogeneous herbaceous vegetation using the following Eq. 1 (FAO, 2015; IPCC, 2006).

1 $$\mathrm{AGC}=\mathrm{AGB}\times0.47$$

where AGC is the aboveground carbon, and AGB is the aboveground biomass.

### Laboratory analysis

Near-infrared spectroscopy (NIRS) is a systematic tool that uses a predetermined wavelength pattern of light (typically 800–2500 nm) to provide a full image of the organic composition of the investigated substance/material (Kilcast, 2013). All the collected samples were analyzed at the Africa laboratory at the University of Pretoria, following a strict and systematic procedure to ensure the most reliable results. Initially, the samples were dried in an oven at 70 °C for 48 h and then ground to a 1-mm particle size using a sieve. Subsequently, the milled samples were analyzed for their chemical composition on a dry matter (DM) basis. Finally, each sample underwent a series of three experiments to derive its foliar biochemical: nitrogen (N) and acid detergent fiber (ADF). These three biochemical were then analyzed using NIRS.

#### Near-infrared spectroscopy (NIRS) analysis and ratio calculation

NIRS is a non-invasive procedure used to measure the proportion of saturated hemoglobin in a target tissue. It depends on two physical principles: differential absorption of “near-infrared light” and the “modified Beer–Lambert law” (Gasser et al., 2022). NIRS devices use light in the near-infrared band (700–900 nm), which can penetrate skin, bone, and connective tissue. The chemical composition of the samples was analyzed using the DA 7250 NIR analyzer, a third-generation diode array NIR tool from “PerkinElmer” designed for quick analysis. The DA 7250 NIR Analyzer can accurately determine nitrogen (N), ADF, and many other parameters and can analyze samples in only 6 s. The tool combines outstanding analytical precision with speed, ease of use, ruggedness, and versatility. It operates in reflectance mode, using a moving rough monochromator to scan the section from “570 to 1850 nm” with an interval of 2 nm (Yi et al., 2017). Then, the C:N:ADF forage ratio for C%, N%, and ADF% was calculated as the proportion between concentrations of these biochemical parameters.

### Remote sensing data acquisition and processing

The study utilized open-free data from Sentinel-1 Synthetic Aperture Radar (SAR) and Sentinel-2 optical sensors (https://code.earthengine.google.com), which offer complementary remotely sensed data for vegetation assessment. The S1 and S2 data cover the study areas and were obtained from Google Earth Engine with a 10% cloud effect. Sentinel-1 and Sentinel-2 data were obtained between the following dates: November–December 2020 (early summer (E.S); July–August 2021 (winter (W), and March 2022 (late summer (L.S)) to represent the full vegetation growing season. All the attained S1 data were from dual polarisation VV/VH, obtained in the “Interferometric Wide swath mode” in the ground range noticed structure (Abdel-Hamid et al., 2020). S2 data comprised 13 bands that cover the spectrum from the visible to “Short-Wave-Infrared (SWIR)” with a high spatial resolution of 10 m (Urban et al., 2021). Vegetation indices were developed from the bands of S2 data for forage C:N:ADF ratio seasonal assessment throughout the study areas (Table 1).

**Table 1:** Vegetation indices and bands used in this study

Index	Bands	Reference
NDVI (Normalized Difference Vegetation Index)	B4, B8	Rouse et al. (1974)
WDVI (Weighted Difference Vegetation Index)	B4, B8	Richardson and Wiegand (1977)
RVI (Ratio Vegetation Index)	B4, B8	Gorai et al. (2014)
MSAVI (Modified Soil Adjusted Vegetation Index)	B4, B8	Qi et al. (1994)
MSAVI2 (Modified Soil Adjusted Vegetation Index 2)	B4, B8	Qi et al. (1994)
NDREI1 (Normalised Difference Red Edge Index 1)	B6, B5	Gitelson and Merzlyak (1994)
NDREI2 (Normalised Difference Red Edge Index 2)	B7, B5	Gitelson and Merzlyak (1994)
MCARI (Modified Chlorophyll Absorption Ratio Index)	B3, B4, B5	Daughtry et al. (2000)
CLRE (Red-edge-band Chlorophyll Index)	B7, B5	Gitelson and Merzlyak (1994)
SATVI (Soil Adjusted Total Vegetation Index)	B4, B11, B12	Marsett et al. (2006)
SLAVI (Specific Leaf Area Vegetation Index)	B4, B8	Lymburner et al. (2000)
SR (Simple Ratio)	B4, B8	Jordan (1969)
SRRE1 _Red-edge1_ (Modified Simple Ratio + Red-edge1)	B4, B8, B5	Sims and Gamon (2002)
SRRE2 _Red-edge2_ (Modified Simple Ratio + Red-edge2)	B4, B8, B6	Sims and Gamon (2002)
NDVIRE_red-edge1_ (Modified Normalized Difference Vegetation Index + Red-edge1)	B8, B5	Gitelson and Merzlyak (1994)
NDVIRE_red-edge2_ (Modified Normalized Difference Vegetation Index + Red-edge2)	B8, B2	Gitelson and Merzlyak (1994)

### Data analysis

The RStudio program, version 4.1.0, was used for conducting all statistical analyses in this study. To gain insight into the overall seasonal quality of forage in the study area, descriptive statistics such as mean, minimum (Min), maximum (Max), and coefficient of variation (CV) were calculated for forage nutrient contents and the C:N:ADF ratio. In order to determine the most effective indicators for estimating chemical components, Pearson’s correlation analysis was done among seasonal individual chemical composition and forage C:N:ADF ratio. A stepwise regression analysis was further applied to validate the observed biochemical and accumulated absorption data. A model was developed for predicting biochemical parameters using field measured accumulated absorption obtained from reflectance. The accuracy of the developed regression model was then evaluated using the cross-validation method, which utilized the caret package and VSURF package applied in R × 64 3.4.0.

### Model development for C:N:ADF ratio estimation

Random decision forest regression is a statistical method used in ecology for predicting the biochemical properties of vegetation. This method is a non-linear collective approach that generates and averages multiple randomized, de-correlated decisions for regression purposes (Hastie et al., 2009). One of the key advantages of this method for ecological studies is the ability to easily include or exclude predictors based on data availability and user requirements. Another benefit is the possibility of including continuous and categorical predictors, such as land use information. Additionally, this method requires fewer user-specified parameters and reduces the risk of overfitting, as well as automatically calculating a variable importance score that assesses the contribution of specific predictors to the final models. This study utilized a RF regression algorithm to estimate forage parameters using unoptimized and optimized combinations of vegetation indices (VIs) and spectral bands, as shown in Table 2.

**Table 2:** The model scenarios were developed using bands and different indices (from S1, S2, and integration)

Model	Sentinel-1 (S1) + Sentinel-2 (S2)
1	Bands + Red-edge + Traditional indices
2	Bands + Red-edge
3	Bands + Traditional indices
4	Red-edge + Traditional indices
5	Bands
6	Red-edge
7	Traditional
8	VH + VV
9	Bands + Red-edge + Traditional indices + VH/VV
10	Bands + Red-edge + VH/VV
11	Red-edge + Traditional indices + VH/VV
12	Bands + Traditional indices + VH/VV
13	Bands + VH/VV
14	Red-edge + VH/VV
15	Traditional + VH/VV

NB: Traditional indices = non-red-edge indices

In this study, model performance was evaluated using cross-validation to determine the coefficient of determination (*R*^2^) as a measure of goodness-of-fit, as well as the root mean square error (RMSE), relative RMSE (%), and mean absolute value of errors (MAE) to assess accuracy. The model’s performance was then evaluated by comparing the differences in *R*^2^ and RMSE between the estimated and measured values of foliage biochemical parameters. Higher *R*^2^ values and lower RMSE and MAE values corresponded to higher accuracy of the model for predicting foliage biochemical parameters. Equations (2) to (5) were used to calculate R2, RMSE, relative RMSE, and MAE, respectively.

2 $${R}^{2}=1-\frac{{\sum }_{i=1}^{n}{(yi- \widetilde{y}i)}^{2}}{{\sum }_{i=1}^{n}{(yi- \overline{y })}^{2}}$$

3 $$\mathrm{RMSE}=\sqrt{\frac1{\overline n}\sum\nolimits_{i=1}^n{(yi-\widetilde yi)}^2}$$

4 $$\mathrm{RRMSE}=\sqrt{\frac{\frac1{\overline n}\sum_{i=1}^n{(yi-\widetilde yi)}^2}{\sum_{i=1}^\text{n}({\widetilde yi)}^2}}$$

5 $$\mathrm{MAE}=\frac1{\overline n}\sum\nolimits_{i=1}^\text{n}\mid\text{y}i-\widetilde yi\mid$$

where *y*_*i*_ is the observed/measured value, *ỹ*_*i*_ is the predicted value, and *ӯ* is the mean of the measured values. *n* = sample size.

## Results

### Seasonal variation of forage in situ

Table 3 illustrates an overview of the seasonal forage concentrations of C, N, and ADF ratio throughout the study area. The results show a seasonal variation among the nutrient concentrations and forage ratios throughout the study area. With high significant concentrations of C and N with low ADF during later summer, followed by early summer and winter with a significant decline in forage N concentration and C:N:ADF ratio. At the same time, C showed no significant difference (*p* < 0.05) between early summer (E.S) and winter (W) seasons as it remained constant and significantly increased during late summer (L.S). The C:N:ADF ratio also shows a seasonal variation with a high mean ratio during L.S (19.35) followed by E.S (11.18) and W with the least mean ratio of 8.28.

**Table 3:** Seasonal distractive statistics of the forage C%, N%, ADF%, and C:N:ADF ratio throughout the study area

Season	Nutrient	Min	mean	Max	StD
E. S	C	0.03	0.14	0.32	0.06
	N	0.59	0.84	1.06	0.12
	ADF	42.44	45.56	48.09	1.63
	C:N:ADF	2.45	11.18	24.85	5.42
W	C	0.03	0.14	0.33	0.07
	N	0.35	0.61	0.95	0.12
	ADF	43.31	47.74	49.99	1.82
	C:N:ADF	3.15	8.28	13.27	3.21
L. S	C	0.11	0.17	0.38	0.04
	N	0.89	1.19	1.57	0.17
	ADF	42.85	44.17	47.74	1.23
	C:N:ADF	11.13	19.35	27	3.56

**Fig. 3:**
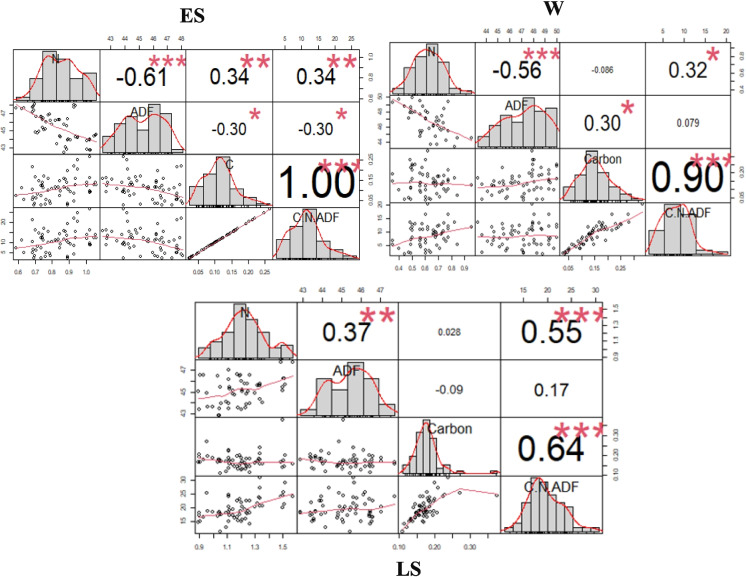
The seasonal Pearson correlations between forage in situ C, N, ADF, and C:N:ADF ratio throughout the study area. Early summer (E.S), winter (W), and later summer (L.S)

The seasonal correlations between the in-situ forage concentrations and the C:N:ADF ratio throughout the study area were calculated (Fig. 3). The in situ forage concentrations were significantly correlated with the C:N:ADF ratio throughout the season with a high correlation among the variables and ratio during E.L, L.S, and W, respectively. A notable strong correlation occurred between the C:N:ADF ratio and C% followed by N%, while ADF showed a weaker correlation throughout the season.

### Seasonal forage C:N:ADF ratio predictive models

Table 4 shows the results of the predictive models with predictor variables after cross-validation to estimate seasonal C:N:ADF ratio using S1, S2, and fusion of S1 and S2. The best seasonal predictive models were selected and recorded using a RF regression algorithm. The results show a relatively weak performance from models derived from individual S1 image data compared to models of individual S2 image data. The best seasonal models for C:N:ADF ratio prediction were observed in synthesizing S1 and S2 data, with the highest *R*^2^ and lowest RMSE. Overall, all further assessments and analyses of seasonal forage C:N:ADF ratio were conducted with the synthesis of S1 and S2 data. Additionally, the red-edges variables such as MCARI, CLRE, and NDREI1 outperformed during the seasonal prediction of forage C:N:ADF ratio models, while only three bands were selected during later summer (B2, B3, B5), while the traditional indices and S1 variables were least selected during the seasonal assessment of the forage C:N:ADF ratio.

**Table 4:** Performance models for predicting seasonal forage C:N:ADF ratio throughout the study area

Season	Models	Variable importance	*R*^2^	RMSE	RRMSE	MAE
E. S	S1	VV	0.51	5.32	33.63	4.36
	S2	NDREI1, SRRE1, WDVI, SLAVI	0.64	4.83	21	3.24
	S1 + S2	NDREI1, CLRE, NDREI2, VH	0.73	4.09	27.70	3.36
W	S1	VV	0.42	3.90	30.57	3.21
	S2	MCARI, CLRE, NDREI1	0.53	3.81	26.44	2.58
	S1 + S2	MCARI, NDREI2, VV, VH	0.68	3.43	27.68	2.81
L.S	S1	VV, VH	0.40	5.06	26.16	3.77
	S2	CLRE, MCARI, NDVIRE1, B5, B2	0.51	5.00	26.05	3.77
	S1 + S2	CLRE, MCARI, NDVIRE1,B5,VV,B3	0.67	4.86	25.95	3.66

*E.S*, early summer; *W*, winter; *L.S*, later-summer; *S1*, Sentinel-1;*S2*, Sentinel-2; *S1* + *S2*, synthesis of Sentinel-1 and Sentinel-2

The selected spatial variables from the best seasonal predictive model were analyzed for their correlation with the forage C:N:ADF ratio across the study area (Table 5). The study revealed varying degrees of correlation between these spatial variables and the forage C:N:ADF ratio in different seasons. During the early summer season, a notably strong and positive significant correlation was observed among the spatial variables and the forage C:N:ADF ratio. In this season, all spatial variables displayed a positive correlation, with correlation coefficients ranging from 0.15 to 0.57. These positive correlations suggest that these spatial variables have a significant impact on the forage C:N:ADF ratio during the early summer. In contrast, during the late summer season (L.S), out of the eight selected variables, only three displayed a significant positive correlation with the forage C:N:ADF ratio. These variables were NDREI1, CLRE, and B5. The other variables did not exhibit a statistically significant relationship with the forage C:N:ADF ratio during this season. It is worth noting that the winter season showed a variable (NDREI2) with a correlation coefficient of 0.11, which was not statistically significant in relation to the forage C:N:ADF ratio. This suggests that during the winter season, the influence of NDREI2 on the forage C:N:ADF ratio was not significant.

**Table 5:** Correlation matrix between the selected or important variables and seasonal C:N:ADF ratio

Season	Variable importance							
E. S	NDREI1	CLRE	NDREI2	SERRE1	WDVI	VV	VH	SLAVI
	0.57***	0.48***	0.46***	0.46***	0.30**	0.21*	0.18*	0.15*
W	MCARI	CLRE	NDREI1	VH	VV	NDREI2		
	0.31**	0.22*	0.20*	0.21*	0.18*	0.11^ns^		
L.S	CLRE	MCARI	NDREI1	VV	B5	VH	B3	B2
	0.46***	0.48***	0.22*	− 0.37**	− 0.30*	− 0.25^ns^	− 0.20^ns^	− 0.20^ns^

NB: Negative correlation ( −); positive correlation ( +);***0.01, **0.05, *0.1; *ns*, non-significant

### Mapping of seasonal forage C:N:ADF ratio

**Fig. 4:**
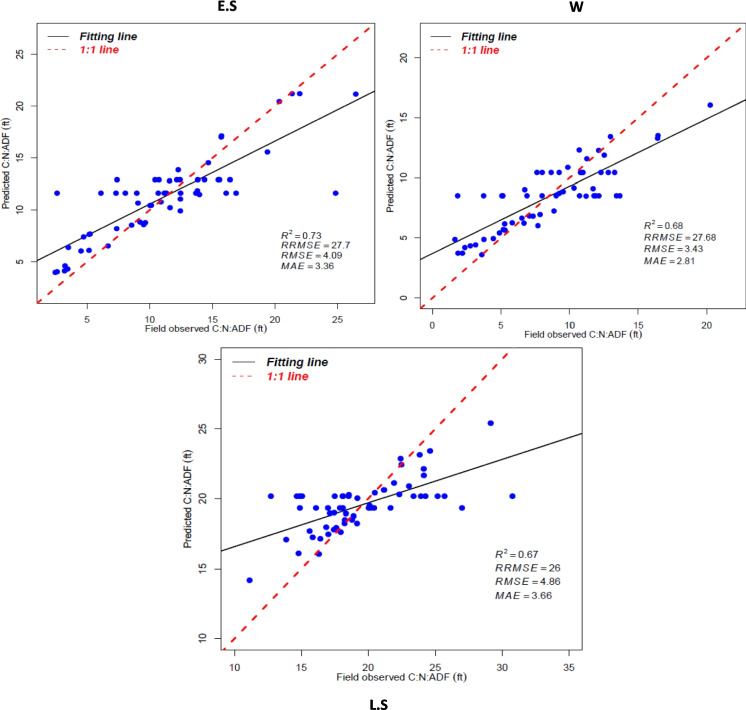
Predicted and measured seasonal forage C:N:ADF ratio plot throughout the study areas

All best models derived from RF using the synthesis of S1 and S2 data were further used to visualize the seasonal distribution of forage C:N:ADF ratio throughout the study areas. The best seasonal predictive forage C:N:ADF ratio models’ distribution is shown in Fig. 4, with a 1:1 scatterplot of predicted against measured ratio values throughout the study area. The seasonal graphical presentation of the C:N:ADF ratio offers the ability to see if the model predictions are equally precise across the entire prediction range. Based on the visual scatterplots, the model was precisely to be used to predict seasonal C:N:ADF ratio throughout the study areas. Several of the predicted seasonal forage C:N:ADF ratio points are closely fit to the measured values.

**Fig. 5:**
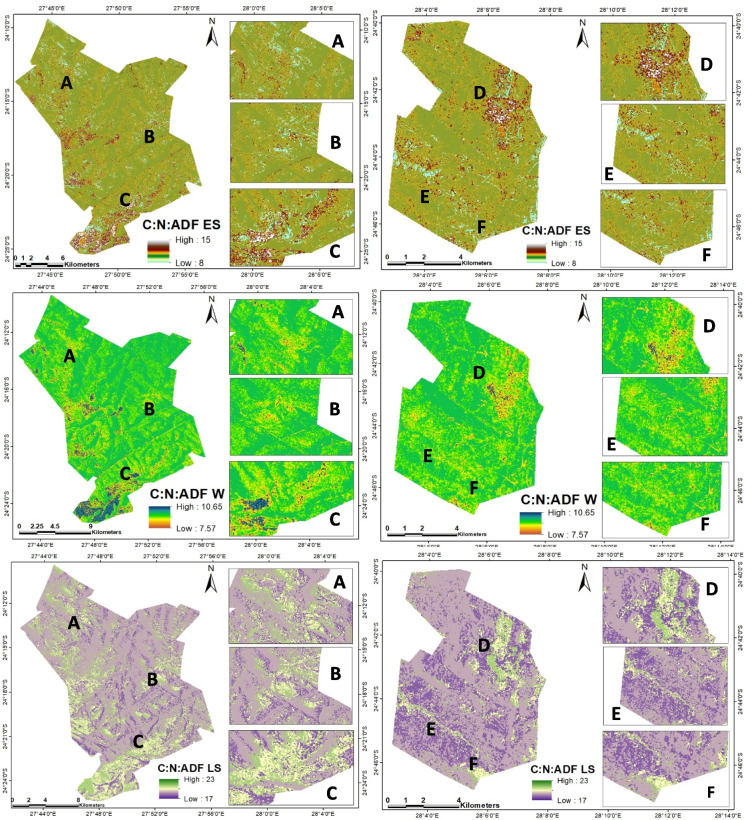
Seasonal spatial distribution of forage C:N:ADF ratio from Sentinel-1 and Sentinel-2 imagery data synthesis. Early summer (E.S), winter (W), and later summer (L.S). Selected or sample areas are marked with A, B, C, D, E, and F, respectively

Figure 5 illustrates the realistic spatial distribution patterns of seasonal forage C:N:ADF ratio throughout the study areas. The variability of the forage C:N:ADF ratio is clear during L.S (ranging from 8.30 to 23.92) followed by E.S (3.96 to 16.26) and less during winter (W) (1.23 to 9.02) of the distribution of forage C:N:ADF ratio in both sites. Additionally, both sites show no significant difference (*p* < 0.05) in the distribution of forage C:N:ADF ratio throughout the season (Table 6). However, HG has a high seasonal forage C:N:ADF ratio distribution compared to WV.

**Table 6:** Descriptive statistics of seasonal distribution and occurrence of forage C:N:ADF ratio throughout the study areas

Area	Season	Min	Max	Mean	StD	CV
WV	E. S	3.96	15.06	6.66	3.20	0.48
	W	1.23	8.15	3.99	1.81	0.45
	L.S	8.30	15.32	12.75	1.86	0.16
	S. E	Yes	Yes	Yes	Yes	Yes
HG	E. S	5.59	16.26	10.72	2.77	0.26
	W	1.61	9.02	5.59	1.86	0.33
	L.S	9.76	23.92	14.38	2.76	0.19
	S. E	Yes	Yes	Yes	Yes	Yes

*WV*, Welgevonden; *HG*, Hoogland

## Discussion

### Impact of the season on forage parameters (C%, N%, ADF%, and C:N:ADF ratio)

The seasonal evaluation of forage carbon (C%), nitrogen (N%), acid detergent fiber (ADF%), and C:N:ADF ratio can offer valuable data for feeding patterns and animal distribution in rangelands.

The distribution and occurrence of the concentration of these parameters largely depend on various factors, such as species composition, maturity stage, and ecological conditions (Aganga et al., 2005; Ravhuhali et al., 2022). This study shows that the season significantly impacts the occurrence and distribution of all biochemical parameters (C%, N%, ADF%, and C:N ratio) across the study areas. Notable variations in all biochemical parameters occur during late summer, while early summer and winter show minimal variation, with no statistical difference and a constant C.

However, the similarities or uniformity in C during early summer and winter can be attributed to plant growth, development, and quality changes during these seasons. The changes in vegetation growth and development during early summer and winter have significant ecological implications for vegetation tissues’ physical and chemical composition. Vegetation may exhibit similar carbon occurrence during early summer and winter due to homogenous biomass production. Vegetation typically experiences active growth and photosynthesis in early summer, resulting in higher carbon uptake. In contrast, plant growth is usually limited or dormant during winter, leading to reduced carbon uptake. Despite these differences in growth activity, the overall carbon levels in the forage may remain relatively constant, resulting in no statistical difference (Du et al., 2020; Singh & Yadava, 1974). Furthermore, as known during early summer, vegetation recovers from winter stress and enters its vegetative growth phase; it has high levels of soluble carbohydrates and low levels of fiber components. This is because the vegetation uses these soluble carbohydrates to support new growth and development. As a result, the fiber components, including cellulose and lignin, are relatively uniform at this time. Similarly, during winter, since vegetation enters a period of inactivity and limited growth, it accumulates more fiber components, such as cellulose and lignin, to provide support and protection against ecological stressors. This is significant as it allows vegetation to survive and thrive in harsh winter conditions (Buxton et al., 1995; Forbes & Watson, 1992; Hasanuzzaman et al., 2013; Ntatsi et al., 2018). This is a similar trend to C% since it is derived from biomass, which is directly proportional to forage fiber content (Popp et al.*,* 2020; Mandal et al., 2007).

Simultaneously, during later summers, there is a noticeable increase in nitrogen (N) concentration, which leads to better digestibility (ADF) and a decrease in carbon content in vegetation across rangelands, as observed by several studies (Evitayani et al., 2004; Islam et al., 2020). This phenomenon can be accredited to a combination of factors, including ideal conditions for vegetation growth and development during the rainy season, which leads to increased nitrogen uptake from the soil and translocation of nitrogen from older plant tissues to new growth (Dubey et al., 2021; Uchida, 2000; Vendramini et al., 2019). Additionally, the increased moisture content in the soil during the rainy season can enhance microbial activity, leading to the breakdown of organic matter and a decrease in fiber content. Changes in species composition during the rainy season, with fast-growing herbaceous species dominating the vegetation, may also contribute to the decrease in fiber content, as these species tend to have lower biomass and fiber content than non-palatable species (Islam et al., 2020). These herbaceous species may also have higher nitrogen content due to their rapid growth and uptake of soil nitrogen (Bot & Benites, 2005; Evitayani et al., 2004; Pontes et al., 2020; Stampfli & Zeiter, 2004). The observed seasonal variation in the C:N:ADF ratio can be explained by the differential effects of C, N, and ADF concentrations on the ratio, as highlighted by recent studies on forage ratios (Gao et al., 2020a; Vethathirri et al., 2021). The foliar C:N:ADF ratio is an important ecological indicator that comprehensively explains plant nutritional status, forage quantity (C%), quality (N%), and digestibility (ADF%) in rangelands. This ratio can help develop effective rangeland management strategies that promote sustainable grazing practices and enhance productivity.

### Seasonal spatial distribution and mapping of C:N:ADF ratio

The study has shown that the foliar C:N:ADF ratio can be accurately estimated using a synthesis of S1 and S2 satellites and RF, which yielded the most effective predictive seasonal models compared to individual satellites. The current study’s findings align with the results of other studies that have highlighted the effectiveness of using a combination of S1 and S2 data for vegetation monitoring. Kaplan and Avdan (2018) found that the fusion of S1 and S2 data outperformed other approaches in monitoring wetlands, while Manakos et al. (2020) utilized a similar fusion of data to overcome unfavorable ecological conditions during vegetation monitoring. This effectiveness can be attributed to the unique features of the S1 and S2 sensors equipped with specialized vegetation monitoring components. These sensors utilize different wavelengths of light and microwave radiation to capture various aspects of vegetation characteristics, such as moisture content, canopy structure, and spectral reflectance. Combining the data from both sensors can provide a more comprehensive view of vegetation health and nutrient status (Shrestha et al., 2021).

The results of this study have noteworthy suggestions for assessing forage quality in rangelands from an ecological perspective. The significant contribution of red-edge-based features to assessing seasonal forage C:N:ADF: ratio highlights the ability of remote sensing technologies for rangeland monitoring and management. Previous research by Vethathirri et al. (2021), Gao et al. (2020a), and Ramoelo et al. (2013) has also highlighted the importance of red-edge variables in predicting forage biochemical parameters, such as N:P and C:N ratios (Arogoundade et al., 2023), which are critical indicators of the nutritional quality and palatability of forage. These findings are particularly relevant to rangeland ecosystems, where forage availability and quality are critical factors affecting grazing herbivores’ health, productivity, and the ecosystem’s ecological sustainability.

However, the insensitivity of red-edge features in comparison to other bands in monitoring biochemical parameters like nitrogen, cellulose, and lignin contents is likely attributed to the sensitivity of the red-edge spectral region to canopy structure, which is influenced by the underlying biochemical composition of the foliage. This suggests that the red-edge-based features can provide insights into the ecological processes driving the spatial and temporal patterns of forage quality in rangelands (Lepine et al., 2016; Xu et al., 2019). This study observed that less selection of bands was associated with their sensitivity during the assessment of biochemical parameters such as N and ratios (Gao et al., 2020a; Ramoelo et al., 2013). On the other hand, this study shows a strong, significant correlation between selected spatial variables and C: N:ADF ratio throughout the season. Previous studies noticed a strong correlation between the spectral absorption features, particularly the red-edge-based variables and vegetation biochemical parameters (Foster et al., 2019; Gao et al., 2020a).

Based on the scatterplots and maps, season significantly impacts the distribution of the forage C:N:ADF ratio throughout the study areas. In the current study, we observed a significant seasonal fluctuation in the C:N:ADF ratio, with the highest values recorded during the late summer, followed by the early summer and the lowest values during winter. This variation was correlated with various factors, such as the plant growth stage and the ecological conditions, including rainfall and temperature. As highlighted by some studies during the late summer, the higher C:N:ADF ratio could be linked to favorable conditions that accelerate metabolic processes, leading to the synthesis and accumulation of nutrients in plant tissues (Arogoundade et al., 2023; Mogashoa et al., 2021). Conversely, the low C:N:ADF ratio during winter could be associated with unfavorable conditions due to low rainfall and temperatures that lead to low vegetation metabolism, reducing nutrient acquisition and storage (Gao et al., 2019, 2020a; Shi et al., 2013). The seasonal variations in the C:N:ADF ratio can be caused by different factors such as the vegetation type, soil fertility, and growth stage (Arogoundade et al., 2023; Mogashoa et al., 2021). Arogoundade et al. (2023) and Adjorlolo et al. (2015) have highlighted each vegetation type consists of different grasses with distinct forage biochemical concentrations, and their response to climate change varies. Therefore, these findings suggest that the forage C:N:ADF ratio can serve as a useful seasonal indicator of nutrient status and physiological responses to changes in ecological conditions in rangelands. This ratio can also be used to estimate nutrient limitation or availability of rangelands throughout the season, as Gao et al. (2019) and Güsewell and Koerselman (2002), suggested for the N:P ratio and Vethathirri et al. (2021) for C:N ratio.

### Nutrient limitation or availability

Based on the recommended values for nutrient limitation by Cech et al. (2008), this study found that during the winter season, there is a limitation of carbon (C), nitrogen (N), and acid detergent fiber (ADF) in the forage. This is shown by foliar C:N:ADF ratio values falling between 1.20 and 9, which suggests that these nutrients are limited during this time. Previous research by Koerselman and Meuleman (1996) has suggested that when the N:P ratios are less than 14, production is limited by N alone, while between 14 and 16, production is co-limited, and above 16, it is limited by phosphorus (P). This study observed co-limitation of N and ADF during winter, as values of forage C:N:ADF ratio were less than 14. The vegetation during the winter season is in its dormant stage with limited growth due to inactive photosynthetic processes, leading to low quality (N) and poor digestibility (ADF). Carbon content in forage tends to remain relatively stable or may even increase during winter. This is because, as vegetation matures, it becomes more lignified, developing stronger structural support to endure stressful conditions. However, this increased lignification leads to lower forage quality and poor digestibility, as the vegetation contains a higher proportion of stems compared to leaves (Buxton & Redfearn, 1997).

In contrast, during the early summer, the observed values of forage C:N:ADF ratios were up to 16, indicating a limitation of C only. During this period, the vegetation is in its transition growth stage, with leaf sheaths and culm internodes beginning to elongate, raising the meristematic tissue to a grazable height. At this stage, nitrogen availability in vegetation tends to be relatively higher, resulting in better forage digestibility. Grasses are most vulnerable during this phase, and heavy grazing can slow down their recovery, depending on the vegetation type and soil fertility (George & Rice, 2020; Owen-Smith, 1994). On the other hand, during the late summer season, this study observed no limitation of C, N, or ADF, as forage C:N:ADF ratio values were above 16, which Koerselman and Meuleman (1996) suggested to be a limited range. Thus, this season is when the vegetation is in equilibrium production, and all vegetation parameters are in optimal production. During this time, carbohydrate and protein reserves are reported to be maximized in stem bases and belowground storage organs (Du et al., 2020; Moore et al., 1991). Generally, the season and growth phases of forage directly impact its nutritional content, which in turn influences animal feed intake and production performance, as observed by Gao et al. (2020a). Therefore, the forage C:N:ADF ratio could provide valuable information to ecologists, resource managers, farmers, and park managers, allowing them to understand which nutrient (C, N, or ADF) is limiting and how this limitation affects the resource selection, availability, and animal distribution in rangelands.

## Conclusions

This study used multispectral Sentinel-1 (S1), Sentinel-2 (S2), and their synthesis remote sensing data to estimate the seasonal distribution and occurrence of forage biochemical parameters of forage C:N:ADF ratio from carbon (C%), nitrogen (N%), and acid detergent fiber (ADF%) map in rangelands (Fig. 5). The most interesting outcome of this research is the discovery of seasonal variations in the forage concentrations of carbon, nitrogen, ADF, and the C:N:ADF ratio throughout the study area. These variations were found to have a significant impact on the forage available for livestock, with different seasons showing varying levels of forage concentrations and ratios. Additionally, the study’s predictive models using satellite data were able to accurately estimate the forage C:N:ADF ratio, providing valuable information for land managers and animal producers in optimizing grazing strategies and animal nutrition. The spatial distribution mapping of the forage C:N:ADF ratio further highlighted the variability of forage quality across different seasons and sites, providing insights into potential management strategies for sustainable animal production.

Therefore, this research makes significant contributions to the field of remote sensing for ecology and rangeland management by demonstrating the potential of satellite data in monitoring and predicting seasonal variations in forage production. By utilizing spectral data from multispectral S1 and S2 satellites, the study was able to develop predictive models for assessing the C:N:ADF ratio of forage, which is a crucial indicator of forage available for animals. This application of remote sensing technology offers a non-invasive and cost-effective method for assessing and monitoring forage available over large spatial scales, offering valuable information for sustainable rangeland management practices. This approach eliminates the need for labor-intensive and time-consuming field sampling and laboratory analysis, providing a quick and reliable method for assessing rangeland status. However, further assessments can be conducted to monitor the seasonal forage C:N:ADF ratio across different rangelands using various methods and explore its economic value from an animal perspective, both at the national and global levels.

## Data Availability

No datasets were generated or analysed during the current study.
